# To test or not to test: Preliminary assessment of normality when comparing two independent samples

**DOI:** 10.1186/1471-2288-12-81

**Published:** 2012-06-19

**Authors:** Justine Rochon, Matthias Gondan, Meinhard Kieser

**Affiliations:** 1 Institute of Medical Biometry and Informatics, University of Heidelberg, Im Neuenheimer Feld 305, 69120, Heidelberg, Germany

**Keywords:** Testing for normality, Student’s *t * test, Mann-Whitney’s *U * test

## Abstract

**Background:**

Student’s two-sample *t * test is generally used for comparing the means of two independent samples, for example, two treatment arms. Under the null hypothesis, the *t * test assumes that the two samples arise from the same normally distributed population with unknown variance. Adequate control of the Type I error requires that the normality assumption holds, which is often examined by means of a preliminary Shapiro-Wilk test. The following two-stage procedure is widely accepted: If the preliminary test for normality is not significant, the *t * test is used; if the preliminary test rejects the null hypothesis of normality, a nonparametric test is applied in the main analysis.

**Methods:**

Equally sized samples were drawn from exponential, uniform, and normal distributions. The two-sample *t * test was conducted if either both samples (Strategy I) or the collapsed set of residuals from both samples (Strategy II) had passed the preliminary Shapiro-Wilk test for normality; otherwise, Mann-Whitney’s *U * test was conducted. By simulation, we separately estimated the conditional Type I error probabilities for the parametric and nonparametric part of the two-stage procedure. Finally, we assessed the overall Type I error rate and the power of the two-stage procedure as a whole.

**Results:**

Preliminary testing for normality seriously altered the conditional Type I error rates of the subsequent main analysis for both parametric and nonparametric tests. We discuss possible explanations for the observed results, the most important one being the selection mechanism due to the preliminary test. Interestingly, the overall Type I error rate and power of the entire two-stage procedure remained within acceptable limits.

**Conclusion:**

The two-stage procedure might be considered incorrect from a formal perspective; nevertheless, in the investigated examples, this procedure seemed to satisfactorily maintain the nominal significance level and had acceptable power properties.

## Background

Statistical tests have become more and more important in medical research [1-3], but many publications have been reported to contain serious statistical errors [4-10]. In this regard, violation of distributional assumptions has been identified as one of the most common problems: According to Olsen [9], a frequent error is to use statistical tests that assume a normal distribution on data that are actually skewed. With small samples, Neville et al. [10] considered the use of parametric tests erroneous unless a test for normality had been conducted before. Similarly, Strasak et al. [7] criticized that contributors to medical journals often failed to examine and report that assumptions had been met when conducting Student’s *t * test.

Probably one of the most popular research questions is whether two independent samples differ from each other. Altman, for example, stated that “most clinical trials yield data of this type, as do observational studies comparing different groups of subjects” ([11], p. 191). In Student’s *t * test, the expectations of two populations are compared. The test assumes independent sampling from normal distributions with equal variance. If these assumptions are met and the null hypothesis of equal population means holds true, the test statistic *T * follows a *t * distribution with *n *_X_ + *n *_Y_ – 2 degrees of freedom:

(1) $$T=\frac{m_{\text{X}}-m_{\text{Y}}}{s\sqrt{\frac{1}{n_{\text{X}}}+\frac{1}{n_{\text{Y}}}}}\text{,}$$

where *m *_X_ and *m *_Y_ are the observed sample means, *n *_X_ and *n *_Y_ are the sample sizes of the two groups, and *s * is an estimate of the common standard deviation. If the assumptions are violated, *T * is compared with the wrong reference distribution, which may result in a deviation of the actual Type I error from the nominal significance level [12,13], in a loss of power relative to other tests developed for similar problems [14], or both. In medical research, normally distributed data are the exception rather than the rule [15,16]. In such situations, the use of parametric methods is discouraged, and nonparametric tests (which are also referred to as distribution-free tests) such as the two-sample Mann–Whitney *U * test are recommended instead [11,17].

Guidelines for contributions to medical journals emphasize the importance of distributional assumptions [18,19]. Sometimes, special recommendations are provided. When addressing the question of how to compare changes from baseline in randomized clinical trials if data do not follow a normal distribution, Vickers, for example, concluded that such data are best analyzed with analysis of covariance [20]. In clinical trials, a detailed description of the statistical analysis is mandatory [21]. This description requires good knowledge about the clinical endpoints, which is often limited. Researchers, therefore, tend to specify alternative statistical procedures in case the underlying assumptions are not satisfied (e.g., [22]). For the *t * test, Livingston [23] presented a list of conditions that must be considered (e.g., normal distribution, equal variances, etc.). Consequently, some researchers routinely check if their data fulfill the assumptions and change the analysis method if they do not (for a review, see [24]).

In a preliminary test, a specific assumption is checked; the outcome of the pretest then determines which method should be used for assessing the main hypothesis [25-28]. For the paired *t * test, Freidlin et al. ([29], p. 887) referred to as “a natural adaptive procedure (…) to first apply the Shapiro-Wilk test to the differences: if normality is accepted, the *t * test is used; otherwise the Wilcoxon signed ranked test is used.” Similar two-stage procedures including a preliminary test for normality are common for two-sample *t * tests [30,31]. Therefore, conventional statistical practice for comparing continuous outcomes from two independent samples is to use a pretest for normality (H_0_: “The true distribution is normal” against H_1_: “The true distribution is non-normal”) at significance level *α *_pre_ before testing the main hypothesis. If the pretest is not significant, the statistic *T * is used to test the main hypothesis of equal population means at significance level *α *. If the pretest is significant, Mann-Whitney’s *U * test may be applied to compare the two groups. Such a two-stage procedure ( Additional file 1) appears logical, and goodness-of-fit tests for normality are frequently reported in articles [32-35].

Some authors have recently warned against preliminary testing [24,36-45]. First of all, theoretical drawbacks exist with regard to the preliminary testing of assumptions. The basic difficulty of a typical pretest is that the desired result is often the acceptance of the null hypothesis. In practice, the conclusion about the validity of, for example, the normality assumption is then implicit rather than explicit: Because insufficient evidence exists to reject normality, normality will be considered true. In this context, Schucany and Ng [41] speak about a “logical problem”. Further critiques of preliminary testing focused on the fact that assumptions refer to characteristics of populations and not to characteristics of samples. In particular, small to moderate sample sizes do not guarantee matching of the sample distribution with the population distribution. For example, Altman ([11], Figure 4.7, p. 60) showed that even sample sizes of 50 taken from a normal distribution may look non-normal. Second, some preliminary tests are accompanied by their own underlying assumptions, raising the question of whether these assumptions also need to be examined. In addition, even if the preliminary test indicates that the tested assumption does not hold, the actual test of interest may still be robust to violations of this assumption. Finally, preliminary tests are usually applied to the same data as the subsequent test, which may result in uncontrolled error rates. For the one-sample *t * test, Schucany and Ng [41] conducted a simulation study of the consequences of the two-stage selection procedure including a preliminary test for normality. Data were sampled from normal, uniform, exponential, and Cauchy populations. The authors estimated the Type I error rate of the one-sample *t * test, given that the sample had passed the Shapiro-Wilk test for normality with a *p * value greater than *α *_pre_. For exponentially distributed data, the conditional Type I error rate of the main test turned out to be strikingly above the nominal significance level and even increased with sample size. For two-sample tests, Zimmerman [42-45] addressed the question of how the Type I error and power are modified if a researcher’s choice of test (i.e., *t * test for equal versus unequal variances) is based on sample statistics of variance homogeneity. Zimmerman concluded that choosing the pooled or separate variance version of the *t * test solely on the inspection of the sample data does neither maintain the significance level nor protect the power of the procedure. Rasch et al. [39] assessed the statistical properties of a three-stage procedure including testing for normality and for homogeneity of the variances. The authors concluded that assumptions underlying the two-sample *t * test should not be pre-tested because “pre-testing leads to unknown final Type I and Type II risks if the respective statistical tests are performed using the same set of observations”. Interestingly, none of the studies cited above explicitly addressed the unconditional error rates of the two-stage procedure as a whole. The studies rather focused on the conditional error rates, that is, the Type I and Type II error of single arms of the two-stage procedure.

In the present study, we investigated the statistical properties of Student’s *t * test and Mann-Whitney’s *U * test for comparing two independent groups with different selection procedures. Similar to Schucany and Ng [41], the tests to be applied were chosen depending on the results of the preliminary Shapiro-Wilk tests for normality of the two samples involved. We thereby obtained an estimate of the conditional Type I error rates for samples that were classified as normal although the underlying populations were in fact non-normal, and vice-versa. This probability reflects the error rate researchers may face with respect to the main hypothesis if they mistakenly believe the normality assumption to be satisfied or violated. If, in addition, the power of the preliminary Shapiro-Wilk test is taken into account, the potential impact of the entire two-stage procedure on the overall Type I error rate and power can be directly estimated.

## Methods

In our simulation study, equally sized samples for two groups were drawn from three different distributions, covering a variety of shapes of data encountered in clinical research. Two selection strategies were examined for the main test to be applied. In Strategy I, the two-sample *t * test was conducted if both samples had passed the preliminary Shapiro-Wilk test for normality; otherwise, we applied Mann-Whitney’s *U * test. In Strategy II, the *t * test was conducted if the residuals $\left(x_{i}-m_{X}),(y_{i}-m_{Y}\right)$from both samples had passed the pretest; otherwise, we used the *U * test. The difference between the two strategies is that, in Strategy I, the Shapiro-Wilk test for normality is separately conducted on raw data from each sample, whereas in Strategy II, the preliminary test is applied only once, i.e. to the collapsed set of residuals from both samples.

Statistical language R 2.14.0 [46] was used for the simulations. Random sample pairs of size *n *_X_ = *n *_Y_ = 10, 20, 30, 40, 50 were generated from the following distributions: (1) exponential distribution with unit expectation and variance; (2) uniform distribution in [0, 1]; and (3) the standard normal distribution. This procedure was repeated until 10,000 pairs of samples had passed the preliminary screening for normality (either Strategy I or II, with *α *_pre_ = .100, .050, .010, .005, or no pretest). For these samples, the null hypothesis *μ *_X_ = *μ *_Y_ was tested against the alternative *μ *_X_ ≠ *μ *_Y_ using Student’s *t * test at the two-sided significance level *α * = .05. The conditional Type I errors rates (left arm of the decision tree in Additional file 1) were then estimated by the number of significant *t * tests divided by 10,000. The precision of the results thereby amounts to maximally ±1% (width of the 95% confidence interval for proportion 0.5). In a second run, sample generation was repeated until 10,000 pairs were collected that had failed preliminary screening for normality (Strategy I or II), and the conditional Type I error was estimated for Mann-Whitney’s *U * test (right part of Additional file 1).

Finally, 100,000 pairs of samples were generated from exponential, uniform, and normal distributions to assess the unconditional Type I error of the entire two-stage procedure. Depending on whether the preliminary Shapiro-Wilk test was significant or not, Mann-Whitney’s *U * test or Student’s *t * test was conducted for the main analysis. The Type I error rate of the entire two-stage procedure was estimated by the number of significant tests (*t * or *U *) and division by 100,000.

## Results

### Strategy I

The first strategy required both samples to pass the preliminary screening for normality to proceed with the two-sample *t * test; otherwise, we used Mann-Whitney’s *U * test. This strategy was motivated by the well-known assumption that the two-sample *t * test requires data within each of the two groups to be sampled from normally distributed populations (e.g., [11]).

**Figure 1:**
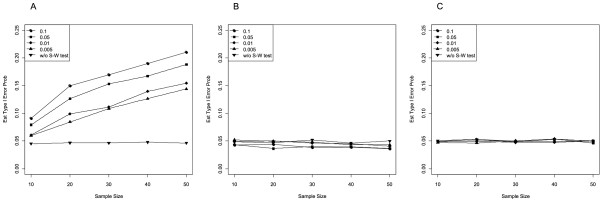
**Estimated Type I error probability of the two-sample *t * test at *α * ** **= .05 after both samples had passed the Shapiro-Wilk test for normality at *α * **_**pre**_ **= .100, .050, .010, .005 (conditional), and without pretest (unconditional).** Samples of equal size from the (**A**) exponential, (**B**) uniform, and (**C**) normal distribution.

Table 1 (left) summarizes the estimated conditional Type I error probabilities of the standard two-sample *t * test (i.e., *t * test assuming equal variances) at the two-sided nominal level *α * = .05 after both samples had passed the Shapiro-Wilk test for normality, as well as the unconditional Type I error rate of the *t * test without a pretest for normality. Figure 1 additionally plots the corresponding estimates if the underlying distribution was either (A) exponential, (B) uniform, or (C) normal. As can be seen from Table 1 and Figure 1, the unconditional two-sample *t * test (i.e., without pretest) was *α *-robust, even if the underlying distribution was exponential or uniform. In contrast, the observed conditional Type I error rates differed from the nominal significance level. For the exponential distribution, the selective application of the two-sample *t * test to pairs of samples that had been accepted as normal led to Type I error rates of the final *t * test that were considerably larger than *α * = .05 (Figure 1A). Moreover, the violation of the significance level increased with sample size and *α *_pre_. For example, for *n * = 30, the observed Type I error rates of the two-sample *t * test turned out to be 10.8% for *α *_pre_ = .005 and even 17.0% for *α *_pre_ = .100, whereas the unconditional Type I error rate was 4.7%. If the underlying distribution was uniform, the conditional Type I error rates declined below the nominal level, particularly as samples became larger and preliminary significance levels increased (Figure 1B). For normally distributed populations, conditional and unconditional Type I error rates roughly followed the nominal significance level (Figure 1C).

**Table 1:** **Left: Estimated Type I error probability of the two-sample *t * test at *α * ** **= .05 after both samples had passed the Shapiro-Wilk test for normality (Strategy I with *α * **_**pre**_ **= .100, .050, .010, .005), and without pretest.—Right: Estimated Type I error of the *U * test for samples that failed testing for normality**

		***t * test**					***U * test**				
	***α ***_**pre**_	***n * = 10**	***n * = 20**	***n * = 30**	***n * = 40**	***n * = 50**	***n * = 10**	***n * = 20**	***n * = 30**	***n * = 40**	***n * = 50**
*Exponential distribution *											
	.100	.091	.150	.170	.190	.210	.050	.048	.051	.050	.051
	.050	.079	.127	.153	.168	.188	.052	.052	.050	.045	.053
	.010	.061	.099	.112	.140	.154	.055	.051	.049	.049	.047
	.005	.060	.085	.108	.127	.144	.060	.047	.052	.049	.048
	Without pretest	.045	.047	.047	.048	.047	.053	.050	.048	.050	.051
*Uniform distribution *											
	.100	.043	.044	.039	.039	.036	.075	.055	.052	.051	.049
	.050	.043	.037	.040	.040	.037	.093	.059	.058	.051	.051
	.010	.049	.050	.046	.045	.041	.168	.111	.074	.060	.057
	.005	.052	.050	.048	.044	.043	.233	.133	.087	.069	.059
	Without pretest	.058	.047	.052	.047	.050	.050	.050	.052	.048	.049
*Normal distribution *											
	.100	.049	.053	.050	.049	.050	.069	.058	.055	.061	.056
	.050	.049	.050	.050	.053	.046	.069	.063	.062	.064	.059
	.010	.050	.050	.047	.048	.051	.090	.081	.073	.072	.074
	.005	.047	.047	.050	.054	.050	.093	.085	.084	.081	.073
	Without pretest	.051	.053	.049	.053	.050	.054	.047	.047	.049	.049

For pairs in which at least one sample had not passed the pretest for normality, we conducted Mann-Whitney’s *U * test. The estimated conditional Type I error probabilities are summarized in Table 1 (right): For exponential samples, only a negligible tendency towards conservative decisions was observed, but samples from the uniform distribution, and, to a lesser extent, samples from the normal distribution proved problematic. In contrast to the pattern observed for the conditional *t * test, however, the nominal significance level was mostly violated in small samples and numerically low significance levels of the pretest (e.g., *α *_pre_ = .005).

### Strategy II

The two-sample *t * test is a special case of a linear model that assumes independent normally distributed errors. Therefore, the normality assumption can be examined through residuals instead of raw data. In linear models, residuals are defined as differences between observed and expected values. In the two-sample comparison, the expected value for a measurement corresponds to the mean of the sample from which it derived, so that the residual simplifies to the difference between the observed value and the sample mean. In regression modeling, the assumption of normality is often checked by the plotting of residuals after parameter estimation. However, this order may be reversed, and formal tests of normality based on residuals may be carried out. In Strategy II, one single Shapiro-Wilk test was applied to the collapsed set of residuals from both samples; thus, in contrast to Strategy I, only one pretest for normality had to be passed.

**Figure 2:**
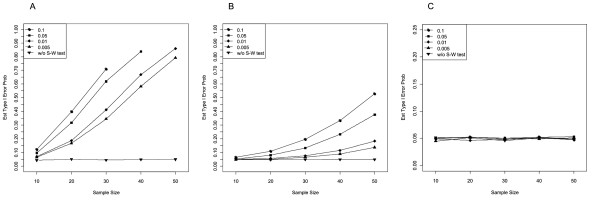
**Estimated Type I error probability of the two-sample *t * test at *α * ** **= .05 after the residuals had passed the Shapiro-Wilk test for normality at *α * **_**pre**_ **= .100, .050, .010, .005 (conditional), and without pretest (unconditional).** Samples of equal size from the (**A**) exponential, (**B**) uniform, and (**C**) normal distribution.

Table 2 (left) and Figure 2 show the estimated conditional Type I error probabilities of the two-sample *t * test at *α * = .05 (two-sided) after residuals had passed the Shapiro-Wilk test for the three different underlying distributions and for different *α *_pre_ levels as well as the corresponding unconditional Type I error rates (i.e., without pretest). For the normal distribution, the conditional and the unconditional Type I error rates were very close to the nominal significance level for all sample sizes and *α *_pre_ levels considered. Thus, if the underlying distribution was normal, the preliminary Shapiro-Wilk test for normality of the residuals did not affect the Type I error probability of the subsequent two-sample *t * test.

**Table 2:** **Left: Estimated Type I error probability of the two-sample *t * test at *α * ** **= .05 for samples that passed testing for normality of the residuals (Strategy II with *α * **_**pre**_ **= .100, .050, .010, .005), and without pretest.—Right: Estimated Type I error for the *U * test for samples that failed testing for normality**

		***t * test**					***U test ***				
	***α ***_**pre**_	***n * = 10**	***n * = 20**	***n * = 30**	***n * = 40**	***n * = 50**	***n * = 10**	***n * = 20**	***n * = 30**	***n * = 40**	***n * = 50**
*Exponential distribution *											
	.100	.122	.398	.709	N/A	N/A	.037	.049	.047	.047	.050
	.050	.096	.317	.611	.839	N/A	.034	.046	.048	.050	.050
	.010	.072	.196	.421	.669	.859	.034	.046	.045	.051	.050
	.005	.064	.162	.347	.583	.792	.036	.040	.047	.044	.048
	Without pretest	.044	.048	.047	.049	.051	.041	.054	.046	.044	.050
*Uniform distribution *											
	.100	.065	.108	.196	.333	.529	.027	.024	.029	.041	.045
	.050	.052	.081	.133	.233	.377	.025	.018	.022	.035	.044
	.010	.051	.057	.076	.116	.184	.036	.011	.013	.022	.031
	.005	.047	.050	.066	.090	.138	.046	.012	.012	.016	.029
	Without pretest	.051	.048	.049	.050	.050	.043	.053	.047	.048	.050
*Normal distribution *											
	.100	.049	.053	.048	.053	.048	.071	.074	.063	.062	.061
	.050	.051	.052	.051	.052	.053	.085	.079	.071	.064	.067
	.010	.049	.046	.048	.051	.049	.120	.107	.087	.073	.073
	.005	.045	.051	.050	.049	.051	.153	.107	.090	.083	.079
	Without pretest	.052	.051	.046	.051	.048	.044	.045	.051	.044	.050

*Note: * N/A, not available because nearly all samples were detected to deviate significantly from the normal distribution.

For the two other distributions, the results were strikingly different. For samples from the exponential distribution, conditional Type I error rates were much larger than the nominal significance level (Figure 2A). For example, at *α *_pre_ = .005, conditional Type I error rates ranged between 6.4% for *n * = 10 up to 79.2% in samples of *n * = 50. For the largest preliminary *α *_pre_ level of .100, samples of *n * = 30 reached error rates above 70%. Thus, the discrepancy between the observed Type I error rate and the nominal *α * was even more pronounced than for Strategy I and increased again with growing preliminary *α *_pre_ and increasing sample size.

Surprisingly and in remarkable contrast to the results observed for Strategy I, samples from the uniform distribution that had passed screening for normality of residuals also led to conditional Type I error rates that were far above 5% (Figure 2B). The distortion of the Type I error rate was only slightly less extreme for the uniform than for the exponential distribution, resulting in error rates up to 50%. The conditional Type I error rate increased again with growing sample size and increasing preliminary significance level of the Shapiro-Wilk test. For example, at *α *_pre_ = .100, conditional Type I error rates were between 6.5% for *n * = 10 and even 52.9% for *n * = 50. Similarly, in samples of *n * = 50, the conditional Type I error rate was between 13.8% for *α *_pre_ = .005 and 52.9% for *α *_pre_ = .100, whereas the Type I error rate without pretest was close to 5.0%.

If the distribution of the residuals was judged as non-normal by the preliminary Shapiro-Wilk test, the two samples were compared by means of Mann-Whitney’s *U * test (Table 2, right). As for Strategy I, the Type I error rate of the conditional *U * test was closest to the nominal *α * for samples from the exponential distribution. For samples from the uniform distribution, the *U * test did not fully exhaust the significance level but showed remarkably anti-conservative behavior for samples drawn from the normal distribution, which was most pronounced in small samples and numerically low *α *_pre_.

### Entire two-stage procedure

Biased decisions within the two arms of the decision tree in Additional file 1 are mainly a matter of theoretical interest, whereas the unconditional Type I error and power of the two-stage procedure reflect how the algorithm works in practice. Therefore, we directly assessed the practical consequences of the entire two-stage procedure with respect to the overall, unconditional, Type I error. This evaluation was additionally motivated by the anticipation that, although the observed conditional Type I error rates of both the main parametric test and the nonparametric test were seriously altered by screening for normality, these results will rarely occur in practice because the Shapiro-Wilk test is very powerful in large samples. Again, pairs of samples were generated from exponential, uniform, and normal distributions. Depending on whether the preliminary Shapiro-Wilk test was significant or not, Mann-Whitney’s *U * test or Student’s *t * test was conducted in the main analysis. Table 3 outlines the estimated unconditional Type I error rates. In line with this expectation, the results show that the two-stage procedure as a whole can be considered robust with respect to the unconditional Type I error rate. This holds true for all three distributions considered, irrespectively of the strategy chosen for the preliminary test.

**Table 3:** **Estimated Type I error probability of the two-stage procedure (Student’s** ** *t * test or Mann-Whitney’s *U * test depending on preliminary Shapiro-Wilk test for normality) for different sample sizes and *α * **_**pre**_

		**Strategy I**					**Strategy II**				
	***α ***_**pre**_	***n * = 10**	***n * = 20**	***n * = 30**	***n * = 40**	***n * = 50**	***n * = 10**	***n * = 20**	***n * = 30**	***n * = 40**	***n * = 50**
*Exponential distribution *											
	.100	.050	.050	.048	.049	.048	.053	.050	.048	.049	.048
	.050	.053	.050	.048	.049	.050	.055	.052	.048	.049	.050
	.010	.054	.054	.048	.049	.050	.054	.054	.048	.049	.050
	.005	.050	.056	.050	.048	.049	.050	.055	.049	.048	.049
*Uniform distribution *											
	.100	.049	.050	.047	.049	.049	.052	.051	.048	.049	.049
	.050	.051	.050	.050	.049	.048	.053	.051	.051	.050	.048
	.010	.051	.050	.051	.050	.051	.051	.051	.052	.051	.051
	.005	.052	.049	.049	.051	.050	.052	.050	.050	.052	.050
*Normal distribution *											
	.100	.050	.052	.052	.051	.052	.051	.052	.053	.051	.051
	.050	.051	.051	.051	.051	.051	.051	.051	.051	.051	.050
	.010	.049	.051	.051	.051	.051	.050	.051	.051	.051	.051
	.005	.051	.050	.049	.050	.050	.051	.050	.049	.050	.050

*Note: * Type I error of the unconditional application of Student’s *t * test and Mann-Whitney’s *U * test is shown in Table 1 and Table 2.

Because the two-stage procedure seemed to keep the nominal significance level, we additionally investigated the corresponding statistical power. To this end, 100,000 pairs of samples were drawn from unit variance normal distributions with means 0.0 and 0.6, from uniform distributions in [0.0, 1.0] and [0.2, 1.2], and from exponential distributions with rate parameters 1.0 and 2.0.

As Table 4 shows, statistical power to detect a shift in two normal distributions corresponds to the weighted sum of the power of the unconditional use of Student’s *t * test and Mann-Whitney’s *U * test. When both samples must pass the preliminary test for normality (Strategy I), the weights correspond to (1 – *α *_pre_)^2^ and 1 – (1 – *α *_pre_)^2^ respectively, which is consistent with the rejection rate of the Shapiro-Wilk test under the normality assumption. For Strategy II, the weights roughly correspond to 1 – *α *_pre_ and *α *_pre_ respectively (a minimal deviation can be expected here because the residuals from the two samples are not completely independent). Similar results were observed for shifted uniform distributions and exponential distributions with different rate parameters: In both distributions, the overall power of the two-stage procedure seemed to lie in-between the power estimated for the unconditional *t * test and the *U * test.

**Table 4:** **Estimated power of the two-stage procedure for different sample sizes and *α * **_**pre**_

		**Strategy I**					**Strategy II**				
		***n * = 10**	***n * = 20**	***n * = 30**	***n * = 40**	***n * = 50**	***n * = 10**	***n * = 20**	***n * = 30**	***n * = 40**	***n * = 50**
*Exponential distributions with rate parameters 1.0 and 2.0 *											
	*U * test only	.224	.443	.612	.743	.835					
	*α *_pre_ = .100	.248	.446	.609	.743	.835	.259	.449	.609	.743	.835
	*α *_pre_ = .050	.254	.451	.610	.744	.835	.261	.454	.610	.744	.835
	*α *_pre_ = .010	.270	.467	.615	.744	.835	.271	.466	.615	.744	.835
	*α *_pre_ = .005	.264	.482	.615	.743	.837	.265	.475	.614	.743	.837
	*t * test only	.240	.518	.721	.847	.919					
*Uniform distributions in [0.0, 1.0] and [0.2, 1.2] *											
	*U * test only	.256	.512	.686	.813	.892					
	*α *_pre_ = .100	.287	.537	.703	.817	.894	.290	.529	.697	.816	.895
	*α *_pre_ = .050	.292	.550	.714	.821	.894	.294	.542	.702	.817	.894
	*α *_pre_ = .010	.295	.558	.740	.848	.908	.295	.559	.729	.835	.900
	*α *_pre_ = .005	.294	.561	.749	.855	.915	.295	.563	.742	.842	.906
	*t * test only	.292	.561	.748	.867	.930					
*Normal distributions with means 0.0 and 0.6 and unit variance *											
	*U * test only	.215	.434	.600	.731	.824					
	*α *_pre_ = .100	.244	.455	.626	.750	.840	.249	.459	.631	.754	.843
	*α *_pre_ = .050	.245	.456	.625	.753	.842	.248	.459	.628	.755	.844
	*α *_pre_ = .010	.244	.455	.629	.756	.842	.245	.455	.630	.756	.842
	*α *_pre_ = .005	.245	.458	.627	.751	.845	.246	.458	.628	.752	.845
	*t * test only	.247	.456	.627	.754	.844					

## Discussion

The appropriateness of a statistical test, which depends on underlying distributional assumptions, is generally not a problem if the population distribution is known in advance. If the assumption of normality is known to be wrong, a nonparametric test may be used that does not require normally distributed data. Difficulties arise if the population distribution is unknown—which, unfortunately, is the most common scenario in medical research. Many statistical textbooks and articles state that assumptions should be checked before conducting statistical tests, and that tests should be chosen depending on whether the assumptions are met (e.g., [22,28,47,48]). Various options for testing assumptions are easily available and sometimes even automatically generated within the standard output of statistical software (e.g., see SAS or SPSS for the assumption of variance homogeneity for the *t * test; for a discussion see [42-45]). Similarly, methodological guidelines for clinical trials generally recommend checking for conditions underlying statistical methods. According to ICH E3, for example, when presenting the results of a statistical analysis, researchers should demonstrate that the data satisfied the crucial underlying assumptions of the statistical test used [49]. Although it is well-known that decision-making after inspection of sample data can lead to altered Type I and Type II error probabilities and sometimes to spurious rejection of the null hypothesis, researchers are often confused or unaware of the potential shortcomings of such two-stage procedures.

### Conditional Type I error rates

We demonstrated the dramatic effects of preliminary testing for normality on the conditional Type I error rate of the main test (see Tables 1 and 2, and Figures 1 and 2). Most of these consequences were qualitatively similar for Strategy I (separate preliminary test for each sample) and Strategy II (preliminary test based on residuals), but quantitatively more pronounced for Strategy II than for Strategy I. On the one hand, the results replicated those found for the one-sample *t * test [41]. On the other hand, our study revealed interesting new findings: Preliminary testing not only affects the Type I error of the *t * test on samples from non-normal distributions but also the performance of Mann-Whitney’s *U * test for equally sized samples from uniform and normal distributions. Since we focused on a two-stage procedure assuming homogenous variances, it can be expected that an additional test for homogeneity of variances should lead to a further distortion of the conditional Type I error rates (e.g., [39,42-45]).

Detailed discussion on potential reasons for the detrimental effects of preliminary tests is provided elsewhere [30,41,50]; therefore, only a global argument is given here: Exponentially distributed variables follow an exponential distribution, and uniformly distributed variables follow a uniform distribution. This trivial statement holds, regardless of whether a preliminary test for normality is applied to the data or not. A sample or a pair of samples is not normally distributed just because the result of the Shapiro-Wilk test suggests it. From a formal perspective, a sample is a set of fixed ‘realizations’; it is not a random variable which could be said to follow some distribution. The preliminary test cannot alter this basic fact; it can only select samples which *appear * to be drawn from a normal distribution. If, however, the underlying population is exponential, the preliminary test selects samples that are not representative of the underlying population. Of course, the Type I error rates of hypotheses tests are strongly altered if they are based on unrepresentative samples. Similarly, if the underlying distribution is normal, the pretest will filter out samples that do not appear normal with probability *α *_pre_. These latter samples are again not representative for the underlying population, so that the Type I error of the subsequent nonparametric test will be equally affected.

In general, the problem is that the distribution of the test statistic of the test of interest depends on the outcome of the pretest. More precisely, errors occurring at the preliminary stage change the distribution of the test statistic at the second stage [38]. As can be seen in Tables 1 and 2, the distortion of the Type I error observed for Strategy I and II is based on at least two different mechanisms. The first mechanism is related to the power of the Shapiro-Wilk test: For the exponential distribution, Strategy I considerably affects the *t * test, but Strategy II does so even more. As both tables show, distortion of the Type I error, if present, is most pronounced in large samples. In line with this result, Strategy II alters the conditional Type I error to a greater extent than Strategy I, probably because in Strategy II, the pretest is applied to the collapsed set of residuals, that is, the pretest is based on a sample twice the size of that used in Strategy I.

To illustrate the second mechanism, asymmetry, we consider the interesting special case of Strategy I applied to samples from uniform distribution. In Strategy I, Mann-Whitney’s *U * test was chosen if the pretest for normality failed in at least one sample. Large violations of the nominal significance level of Mann-Whitney’s *U * test were observed for small samples and numerically low significance levels for the pretest (23.3% for *α *_pre_ = .005 and *n * = 10). At *α *_pre_ = .005 and *n * = 10, the Shapiro-Wilk test has low power, so that only samples with extreme properties will be identified. In general, however, samples from the uniform distribution do not have extreme properties, such that, in most cases, only one member of the sample pair will be sufficiently extreme to be detected by the Shapiro-Wilk test. Consequently, pairs of samples are selected by the preliminary test for which one member is extreme and the other member is representative; the main significance test will then indicate that the samples differ indeed. For these pairs of samples, the Shapiro-Wilk test and the Mann–Whitney *U * test essentially yield the same result because they test similar hypotheses. In contrast, in Strategy II, the pretest selected pairs of samples for which the set of residuals (i.e., the two samples shifted over each other) appeared non-normal. This result mostly corresponds to the standard situation in nonparametric statistics, so that the conditional Type I error rate of Mann-Whitney’s *U * test applied to samples from uniform distribution was unaffected by the asymmetry mechanism.

### Type I error and power of the entire two-stage procedure

On the one hand, our study showed that conditional Type I error rates may heavily deviate from the nominal significance level (Tables 1 and 2). On the other hand, direct assessment of the unconditional Type I error rate (Table 3) and power (Table 4) of the two-stage procedure suggests that the two-stage procedure as a whole has acceptable statistical properties. What might be the reason for this discrepancy? To assess the consequences of preliminary tests for the entire two-stage procedure, the power of the pretest needs to be taken into account,

(2) $$\begin{array}{c} \text{P}\left(\text{TypeIerror}\right)=\text{P}\left(\text{TypeIerror}\cap \text{Pretest}n.s.\right)+\text{P}\left(\text{TypeIerror}\cap \text{Pretest}sig.\right)\\ =\text{P}\left(\text{TypeIerror}|\text{Pretest}n.s.\right)\times \text{P}\left(\text{Pretest}n.s.\right)\\ +\text{P}\left(\text{TypeIerror}|\text{Pretest}sig.\right)\times \text{P}\left(\text{Pretest}sig.\right)\text{,} \end{array}$$

with P(Type I error | Pretest *n.s. *) denoting the conditional Type I error rate of the *t * test (Tables 1 and 2 left), P(Type I error | Pretest *sig. *) denoting the conditional Type I error rate of the *U * test (Tables 1 and 2 right), and P(Pretest *sig. *) and P(Pretest *n.s. *) denoting the power and 1 – power of the pretest for normality. In Strategy I, P(Pretest *sig. *) corresponds to the probability to reject normality for at least one of the two samples, whereas in Strategy II, it is the probability to reject the assumption of normality of the residuals from both samples.

For the *t * test, unacceptable rates of false decisions due to selection effects of the preliminary Shapiro-Wilk test occur for large samples and numerically high significance levels *α *_pre_ (e.g., left column in Table 2). In these settings, however, the Shapiro-Wilk test detects deviations from normality with nearly 100% power, so that the Student’s *t * test is practically never used. Instead, the nonparametric test is used that seems to protect the Type I error for those samples. This pattern of results holds for both Strategy I and Strategy II. Conversely, it was demonstrated above that Mann-Whitney’s *U * test is biased for normally distributed data if the sample size is low and the preliminary significance level is strict (e.g., *α *_pre_ = .005, right columns of Tables 1 or 2). For samples from normal distribution, however, deviation from normality is only rarely detected at *α *_pre_ = .005, so that the consequences for the overall Type I error of the entire two-stage procedure are again very limited.

A similar argument holds for statistical power: For a given alternative, the overall power of the two-stage procedure corresponds, by construction, to the weighted sum of the conditional power of the *t * test and *U * test. When populations deviate only slightly from normality, the pretest for normality has low power, and the power of the two-stage procedure will tend towards the unconditional power of Student’s *t * test; this fact only does not hold in those rare cases in which the preliminary test indicates non-normality, so that the slightly less powerful Mann–Whitney *U * test is applied. When the populations deviate considerably from normality, the power of the Shapiro-Wilk test is high for both strategies, and the overall power of the two-stage procedure will tend towards the unconditional power of Mann-Whitney’s *U * test.

Finally, it should be emphasized that the conditional Type I error rates shown in Tables 1 and 2 correspond to the rather unlikely scenario in which researchers would continue sampling until the assumptions are met. In contrast, the unconditional Type I error and power of the two-stage procedure are most relevant because in practice, researchers do not continue sampling until they obtain normality. Researchers who do not know in advance whether the underlying population distribution is normal, usually base their decision on the samples obtained. If by chance a sample from a non-normal distribution happens to look normal, the researcher could falsely assume that the normality assumption holds. However, this chance is rather low because of the high power of the Shapiro-Wilk test, particularly for larger sample sizes.

## Conclusions

From a formal perspective, preliminary testing for normality is incorrect and should therefore be avoided. Normality has to be established for the populations under consideration; if this is not possible, “support for the assumption of normality must come from extra-data sources” ([30], p. 7). For example, when planning a study, assumptions may be based on the results of earlier trials [21] or pilot studies [36]. Although often limited in size, pilot studies could serve to identify substantial deviations from normality. From a practical perspective, however, preliminary testing does not seem to cause much harm, at least for the cases we have investigated. The worst that can be said is that preliminary testing is unnecessary: For large samples, the *t * test has been shown to be robust in many situations [51-55] (see also Tables 1 and 2 of the present paper) and for small samples, the Shapiro-Wilk test lacks power to detect deviations from normality. If the application of the *t * test is doubtful, the unconditional use of nonparametric tests seems to be the best choice [56].

## Competing interests

The authors declare that they have no competing interests.

## Authors’ contributions

All authors jointly designed the study. JR carried out the simulations, MG assisted in the simulations and creation of the figures. JR and MG drafted the manuscript. MK planned the study and finalized the manuscript. All authors read and approved the final manuscript.

## Pre-publication history

The pre-publication history for this paper can be accessed here:

http://www.biomedcentral.com/1471-2288/12/81/prepub

## Supplementary Material

Additional file 1 Two-stage procedure including a preliminary test for normality. Click here for file

## References

[B1] Altman DG Statistics in medical journals Stat Med 1982 1 59 71 10.1002/sim.4780010109 7187083

[B2] Altman DG Statistics in medical journals: Developments in the 1980s Stat Med 1991 10 1897 1913 10.1002/sim.4780101206 1805317

[B3] Altman DG Statistics in medical journals: Some recent trends Stat Med 2000 19 3275 3289 10.1002/1097-0258(20001215)19:23<3275::AID-SIM626>3.0.CO;2-M 11113959

[B4] Glantz SA Biostatistics: How to detect, correct and prevent errors in medical literature Circulation 1980 61 1 7 10.1161/01.CIR.61.1.1 7349923

[B5] Pocock SJ Hughes MD Lee RJ Statistical problems in the reporting of clinical trials—A survey of three medical journals N Engl J Med 1987 317 426 432 10.1056/NEJM198708133170706 3614286

[B6] Altman DG Poor-quality medical research: What can journals do? JAMA 2002 287 2765 2767 10.1001/jama.287.21.2765 12038906

[B7] Strasak AM Zaman Q Marinell G Pfeiffer KP Ulmer H The use of statistics in medical research: A comparison of The New England Journal of Medicine and Nature Medicine Am Stat 2007 61 47 55 10.1198/000313007X170242

[B8] Fernandes-Taylor S Hyun JH Reeder RN Harris AHS Common statistical and research design problems in manuscripts submitted to high-impact medical journals BMC Res Notes 2011 4 304 10.1186/1756-0500-4-304 21854631 PMC3224575

[B9] Olsen CH Review of the use of statistics in Infection and Immunity Infect Immun 2003 71 6689 6692 10.1128/IAI.71.12.6689-6692.2003 14638751 PMC308915

[B10] Neville JA Lang W Fleischer AB Errors in the Archives of Dermatology and the Journal of the American Academy of Dermatology from January through December 2003 Arch Dermatol 2006 142 737 740 10.1001/archderm.142.6.737 16785376

[B11] Altman DG Practical Statistics for Medical Research 1991 Chapman and Hall, London

[B12] Cressie N Relaxing assumptions in the one sample t-test Aust J Stat 1980 22 143 153 10.1111/j.1467-842X.1980.tb01161.x

[B13] Ernst MD Permutation methods: A basis for exact inference Stat Sci 2004 19 676 685 10.1214/088342304000000396

[B14] Wilcox RR How many discoveries have been lost by ignoring modern statistical methods? Am Psychol 1998 53 300 314

[B15] Micceri T The unicorn, the normal curve, and other improbable creatures Psychol Bull 1989 105 156 166

[B16] Kühnast C Neuhäuser M A note on the use of the non-parametric Wilcoxon-Mann–Whitney test in the analysis of medical studies Ger Med Sci 2008 6 2 5 PMC2703264 19675730

[B17] New England Journal of Medicine: Guidelines for manuscript submission. (Retrieved from http://www.nejm.org/page/author-center/manuscript-submission); 2011

[B18] Altman DG Gore SM Gardner MJ Pocock SJ Statistics guidelines for contributors to medical journals Br Med J 1983 286 1489 1493 10.1136/bmj.286.6376.1489 6405856 PMC1547706

[B19] Moher D Schulz KF Altman DG for the CONSORT Group The CONSORT statement: Revised recommendations for improving the quality of reports of parallel-group randomized trials Ann Intern Med 2001 134 657 662 11304106 10.7326/0003-4819-134-8-200104170-00011

[B20] Vickers AJ Parametric versus non-parametric statistics in the analysis of randomized trials with non-normally distributed data BMC Med Res Meth 2005 5 35 10.1186/1471-2288-5-35 PMC1310536 16269081

[B21] ICH E9 Statistical principles for clinical trials 1998 International Conference on Harmonisation, London, UK

[B22] Gebski VJ Keech AC Statistical methods in clinical trials Med J Aust 2003 178 182 184 12580749

[B23] Livingston EH Who was Student and why do we care so much about his t-test? J Surg Res 2004 118 58 65 10.1016/j.jss.2004.02.003 15093718

[B24] Shuster J Diagnostics for assumptions in moderate to large simple trials: do they really help? Stat Med 2005 24 2431 2438 10.1002/sim.2175 15977289

[B25] Meredith WM Frederiksen CH McLaughlin DH Statistics and data analysis Annu Rev Psychol 1974 25 453 505 10.1146/annurev.ps.25.020174.002321

[B26] Bancroft TA On biases in estimation due to the use of preliminary tests of significance Ann Math Statist 1944 15 190 204 10.1214/aoms/1177731284

[B27] Paull AE On a preliminary test for pooling mean squares in the analysis of variance Ann Math Statist 1950 21 539 556 10.1214/aoms/1177729750

[B28] Gurland J McCullough RS Testing equality of means after a preliminary test of equality of variances Biometrika 1962 49 403 417

[B29] Freidlin B Miao W Gastwirth JL On the use of the Shapiro-Wilk test in two-stage adaptive inference for paired data from moderate to very heavy tailed distributions Biom J 2003 45 887 900 10.1002/bimj.200390056

[B30] Easterling RG Anderson HE The effect of preliminary normality goodness of fit tests on subsequent inference J Stat Comput Simul 1978 8 1 11 10.1080/00949657808810243

[B31] Pappas PA DePuy V An overview of non-parametric tests in SAS: When, why and how Proceeding of the. SouthEast SAS Users Group Conference (SESUG 2004): Paper TU04 2004 Miami, FL, SouthEast SAS Users Group 1 5

[B32] Bogaty P Dumont S O’Hara G Boyer L Auclair L Jobin J Boudreault J Randomized trial of a noninvasive strategy to reduce hospital stay for patients with low-risk myocardial infarction J Am Coll Cardiol 2001 37 1289 1296 10.1016/S0735-1097(01)01131-7 11300437

[B33] Holman AJ Myers RR A randomized, double-blind, placebo-controlled trial of pramipexole, a dopamine agonist, in patients with fibromyalgia receiving concomitant medications Arthritis Rheum 2005 53 2495 2505 16052595 10.1002/art.21191

[B34] Lawson ML Kirk S Mitchell T Chen MK Loux TJ Daniels SR Harmon CM Clements RH Garcia VF Inge TH One-year outcomes of Roux-en-Y gastric bypass for morbidly obese adolescents: a multicenter study from the Pediatric Bariatric Study Group J Pediatr Surg 2006 41 137 143 10.1016/j.jpedsurg.2005.10.017 16410123

[B35] Norager CB Jensen MB Madsen MR Qvist N Laurberg S Effect of darbepoetin alfa on physical function in patients undergoing surgery for colorectal cancer Oncology 2006 71 212 220 10.1159/000106071 17641543

[B36] Shuster J Student t-tests for potentially abnormal data Stat Med 2009 28 2170 2184 10.1002/sim.3581 19326398 PMC3666168

[B37] Schoder V Himmelmann A Wilhelm KP Preliminary testing for normality: Some statistical aspects of a common concept Clin Exp Dermatol 2006 31 757 761 10.1111/j.1365-2230.2006.02206.x 17040259

[B38] Wells CS Hintze JM Dealing with assumptions underlying statistical tests Psychol Sch 2007 44 495 502 10.1002/pits.20241

[B39] Rasch D Kubinger KD Moder K The two-sample t test: pretesting its assumptions does not pay Stat Papers 2011 52 219 231 10.1007/s00362-009-0224-x

[B40] Zimmerman DW A simple and effective decision rule for choosing a significance test to protect against non-normality Br J Math Stat Psychol 2011 64 388 409 10.1348/000711010X524739 21973093

[B41] Schucany WR Ng HKT Preliminary goodness-of-fit tests for normality do not validate the one-sample student t Commun Stat Theory Methods 2006 35 2275 2286 10.1080/03610920600853308

[B42] Zimmerman DW Some properties on preliminary tests of equality of variances in the two-sample location problem J Gen Psychol 1996 123 217 231 10.1080/00221309.1996.9921274

[B43] Zimmerman DW Invalidation of parametric and nonparametric statistical tests by concurrent violation of two assumptions J Exp Educ 1998 67 55 68 10.1080/00220979809598344

[B44] Zimmerman DW Conditional probabilities of rejecting H0 by pooled and separate-variances t tests given heterogeneity of sample variances Commun Stat Simul Comput 2004 33 69 81 10.1081/SAC-120028434

[B45] Zimmerman DW A note on preliminary tests of equality of variances Br J Math Stat Psychol 2004 57 173 181 10.1348/000711004849222 15171807

[B46] R Development Core Team R: A language and environment for statistical computing 2011 R Foundation for Statistical Computing, Vienna, Austria

[B47] Lee AFS Armitage P, Colton T Student’s t statistics Encyclopedia of Biostatistics 2005 2 Wiley, New York

[B48] Rosner B Fundamentals of Biostatistics 1990 3 PWS-Kent, Boston

[B49] ICH E3 Structure and content of clinical study reports 1995 International Conference on Harmonisation, London, UK

[B50] Rochon J Kieser M A closer look at the effect of preliminary goodness-of-fit testing for normality for the one-sample t-test Br J Math Stat Psychol 2011 64 410 426 10.1348/2044-8317.002003 21973094

[B51] Armitage P Berry G Matthews JNS Statistical Methods in Medical Research 2002 Blackwell, Malden, MA

[B52] Boneau CA The effects of violations underlying the t test Psychol Bull 1960 57 49 64 13802482 10.1037/h0041412

[B53] Box GEP Non-normality and tests of variances Biometrika 1953 40 318 335

[B54] Rasch D Guiard V The robustness of parametric statistical methods Psychology Science 2004 46 175 208

[B55] Sullivan LM D’Agostino RB Robustness of the t test applied to data distorted from normality by floor effects J Dent Res 1992 71 1938 1943 10.1177/00220345920710121601 1452898

[B56] Akritas MG Arnold SF Brunner E Nonparametric hypotheses and rank statistics for unbalanced factorial designs J Am Stat Assoc 1997 92 258 265 10.1080/01621459.1997.10473623

