# Supplementary figures and images for: To test or not to test: Preliminary assessment of normality when comparing two independent samples
Source: BMC Med Res Methodol. 2012 Jun 19;12:81. doi: 10.1186/1471-2288-12-81 (PMC3444333; doi:10.1186/1471-2288-12-81)

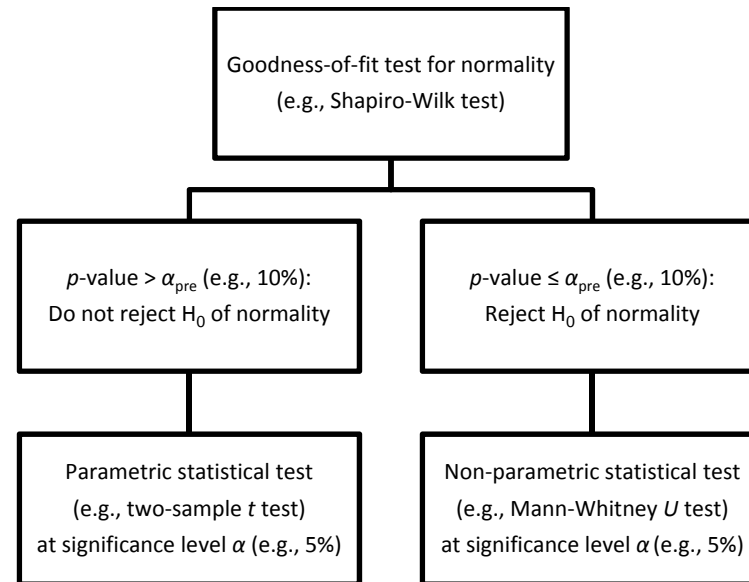

Supplement: Additional file 1 — Two-stage procedure including a preliminary test for normality. [file 1471-2288-12-81-S1.pdf]
